# Using Manifold Learning for Atlas Selection in Multi-Atlas Segmentation

**DOI:** 10.1371/journal.pone.0070059

**Published:** 2013-08-02

**Authors:** Albert K. Hoang Duc, Marc Modat, Kelvin K. Leung, M. Jorge Cardoso, Josephine Barnes, Timor Kadir, Sébastien Ourselin, for the Alzheimer’s Disease Neuroimaging Initiative

**Affiliations:** 1 Centre for Medical Image Computing, University College London, London, United Kingdom; 2 Dementia Research Centre, University College London, London, United Kingdom; 3 Mirada Medical, Oxford, United Kingdom; University of Manchester, United Kingdom

## Abstract

Multi-atlas segmentation has been widely used to segment various anatomical structures. The success of this technique partly relies on the selection of atlases that are best mapped to a new target image after registration. Recently, manifold learning has been proposed as a method for atlas selection. Each manifold learning technique seeks to optimize a unique objective function. Therefore, different techniques produce different embeddings even when applied to the same data set. Previous studies used a single technique in their method and gave no reason for the choice of the manifold learning technique employed nor the theoretical grounds for the choice of the manifold parameters. In this study, we compare side-by-side the results given by 3 manifold learning techniques (Isomap, Laplacian Eigenmaps and Locally Linear Embedding) on the same data set. We assess the ability of those 3 different techniques to select the best atlases to combine in the framework of multi-atlas segmentation. First, a leave-one-out experiment is used to optimize our method on a set of 110 manually segmented atlases of hippocampi and find the manifold learning technique and associated manifold parameters that give the best segmentation accuracy. Then, the optimal parameters are used to automatically segment 30 subjects from the Alzheimer’s Disease Neuroimaging Initiative (ADNI). For our dataset, the selection of atlases with Locally Linear Embedding gives the best results. Our findings show that selection of atlases with manifold learning leads to segmentation accuracy close to or significantly higher than the state-of-the-art method and that accuracy can be increased by fine tuning the manifold learning process.

## Introduction

Multi-atlas segmentation is an automated segmentation method that shows good robustness and accuracy in segmenting various anatomical structures [1]–[4]. In this framework, a segmentation of a target image is obtained through the propagation and fusion of multiple atlas images by mean of registration. As demonstrated by [5], propagation of atlases similar to the target image significantly improves the quality of the segmentation. As a result, it is crucial to develop strategies for selecting the best atlases in the framework of multi-atlas segmentation in order to achieve optimal accuracy.

Several approaches for atlas selection have been proposed over the past few years [2], [3], [5]–[11]. For instance, in the multiple-atlas propagation and segmentation method (MAPS) [7], the most similar atlases are selected based on intensity similarity after rigid registration. In [12], manifold learning is used to select atlases which are located in the neighbourhood of the target on the manifold. This novel approach gives promising results. However, some aspects in that study have not been investigated thoroughly such as the type of manifold learning or optimal manifold parameters. Therefore, our paper investigates further the usage of manifold learning for atlas selection in the framework of multi-atlas segmentation.

Manifold learning has been successfully used in multiple medical imaging applications including segmentation [13], registration [14], [15], classification [16] and statistical population analysis [17], [18]. The most popular manifold learning techniques used in medical imaging are Isomap [19], Locally Linear Embedding (LLE) [20] and Laplacian Eigenmaps (LEM) [21]. For instance, Laplacian Eigenmaps is used by [22] to reduce the computational complexity in multi-modal registration and by [23] for biomarker discovery in MR imaging. Isomap is used by [14] to tackle the problem of performing large deformation registration and by [24] to parametrize cardiac MRI images. [25] investigates the detection of seizures in EEG signals with Locally Linear Embedding.

Each manifold learning technique attempts to preserve a different geometrical property of the underlying manifold. Isomap is a global approach that attempts to preserve pairwise metrics. In contrast, LLE and LEM aim to preserve the local geometry of the data. Since each manifold learning technique is associated with a different objective function, it is legitimate to assume that, for a given data set, the associated embeddings are also different. Previous studies [14], [22]–[25] only included a single technique in their design and manifold parameters appeared to have been chosen arbitrarily. To our knowledge, no studies have investigated (i) the effect of different manifold learning algorithms and (ii) the computation of optimal manifold parameters for a given application. This paper addresses these two points. In the context of atlas selection for multi-atlas segmentation, we investigate the appropriate choice of manifold learning technique and manifold parameters that result in optimal atlas selection and subsequently achieve optimal segmentation accuracy.

## Methods

### 1.1 Ethics Statement

This study was conducted in accordance with the ethical standards stated in the Declaration of Helsinki using publicly available imaging data.

### 1.2 Overview

This paper aims to qualitatively and quantitatively assess the selection of atlases to combine in the framework of multi-atlas segmentation using 3 different manifold learning techniques. We consider Isomap [19], Locally Linear Embedding (LLE) [20] and Laplacian Eigenmaps (LEM) [21] since those techniques are the most widely used in medical imaging.

Our method can be summarized in 3 steps. First, a low-dimensional manifold is learned from the space spanned by the set of atlases using the 3 different techniques (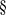 1.3). The neighbourhood relationship on the manifold is derived from non-rigid transformations that align atlases to each other in the high-dimensional space (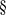 1.4). Second, a new target image is embedded onto the previously computed manifold by means of the out-of-sample extension [26] (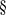 1.5). Third, the target image is segmented using atlases that are within its vicinity on the manifold (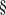 1.6).

For each manifold learning technique, we investigate the effects of (i) the number of dimensions of the resulting embedding, (ii) the number of neighbours used to build the 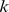-nearest neighbour graph in the high-dimensional space, and (iii) the number of atlases used during the combination process.

An atlas data set composed of 110 manually segmented images of hippocampi from the MIRIAD public data set (www.ucl.ac.uk/drc/research/miriad) is used to optimize each manifold learning technique on a leave-one-out experiment (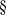 2.1). Segmentation accuracy is then validated on an independent set of 30 manually segmented images from the Alzheimer’s Disease Neuroimaging Initiative (ADNI, www.loni.ucla.edu/ADNI/) (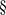 2.2). The MIRIAD data set is described in 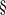 1.7. The ADNI data set is described in 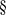 1.8.

### 1.3 Manifold Learning

Given a set of 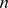 atlases 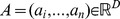, the goal is to identify atlases that are most similar to a target image 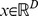 using manifold learning. It has been suggested that the set of brain images has an intrinsic dimensionality meaning that points in data set 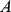 and image 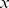 are lying on or near a manifold with dimensionality 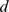 which is embedded in the 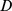-dimensional space [17]. By using manifold learning, data set 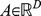 is transformed into a new dataset 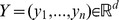 with 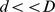, while preserving the non-linear geometry and neighbourhood information of the high-dimensional data in the low-dimensional space. The atlases that are nearest to 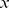 are identified on the low-dimensional manifold and used for segmentation.

Variation in brain images is best described by non-linear dimensionality reduction models compared to linear ones like Principal Component Analysis (PCA) or Multi-Dimensional Scaling (MDS) [17]. In our study, low-dimensional embeddings are computed with 3 different non-linear techniques: Isomap [19], Locally Linear Embedding (LLE) [20] and Laplacian Eigenmaps (LEM) [21]. The differences between those 3 techniques are emphasized by their unique objective functions. For Isomap, the objective function is:
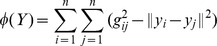
where 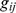 represents the geodesic distance between 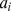 and 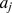 in the high-dimensional space. For LLE, the objective function is:



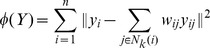
where 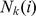 are the 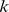-nearest neighbours of 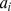 and weight 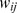 is the contribution of 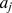 in reconstructing 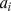 in the high-dimensional space. As demonstrated by [20], the optimal weights 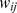 are obtained through minimization by solving a least-squares problem.

Finally, the objective function associated with LEM is:
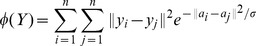



All 3 techniques require the construction of a connected graph in the high-dimensional space using the 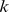-nearest neighbour algorithm. The number of neighbours used to build this connected graph is defined as 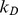.

Unlike PCA, the embedding produced by these techniques is a function of a metric which determines the 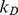-nearest neighbours in the high-dimensional space and subsequently the neighbouring images on the low-dimensional manifold. We use the metric presented in 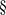 1.4 to find those 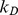-nearest neighbours.

### 1.4 Distance between Pairs of Images

We derive the metric from the method presented by [27]. An atlas 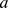 and target image 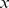 are similar when the non-rigid transformation that aligns them produces a small deformation. Similarity is based on the displacement field 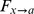 of the non-rigid transformation 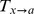. In order to avoid the computational load of performing registrations between all atlases and every new unseen target image, an average atlas 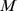 is built from the atlases in the data set using the iterative groupwise registration scheme described by [28]. This enables 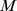 to lie near the center of the space of all atlases. From the average atlas 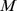, a displacement field 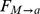 (resp. 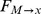) is derived from the non-rigid transformation 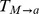 (resp. 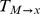) for each atlas 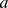 (resp. new target 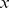). The similarity is then evaluated with:
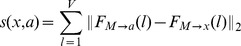
where 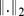 is the L2 norm and 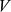 is the number of voxels in each atlas.

In this framework, the similarity between 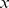 and any atlases 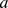 can be evaluated by registering 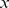 to 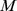. Since 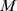 lies near the center of the space of all atlases, the manifold resulting from the approximation of 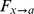 with 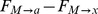 minimizes the error in estimating the neighbourhood relationship when compared to the manifold resulting from the direct computation of 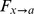.

The non-rigid transformation 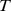 is performed using an efficient implementation [29] of the free-form deformation algorithm [30]. The transformation model is parameterized using a cubic B-Spline scheme and the transformation 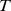 is driven by the normalised mutual information.

### 1.5 Extending a Manifold with a New Target Image 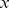


For Isomap, LLE and LEM, the out-of-sample extension is performed using the Nyström approximation [26]. Experiments on real high-dimensional data have demonstrated the accuracy of out-of-sample extension in positioning an out-of-sample point on a low-dimensional manifold [26]. The metric presented in 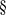 1.4 is also used for extending the manifold.

Since the low-dimensional manifold is embedded in a Euclidean space, the L2 distance is used to determine the 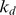-nearest neighbours of 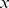 on the manifold. Those 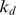-nearest neighbours are subsequently used for label propagation.

### 1.6 Segmentation by Fusion Strategy

STAPLE [31] is used to combine multiple segmentations generated from the most similar atlases. We found in our previous study [7] that STAPLE gives better results compared to a voting rule or shape-based averaging method when using the MIRIAD data set. It simultaneously computes a probabilistic estimate of the true segmentation and a measure of the performance level (sensitivity and specificity) represented by each segmentation in an expectation-maximization framework. An iterative Markov random field optimized with mean field approximation is used to provide spatial consistency in the probabilistic estimate of neighbouring voxels. The STAPLE algorithm is solved only in the non-consensus area in order to reduce bias as suggested by [28]. We denote by 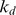 the number of atlases used for label propagation.

### 1.7 Atlas Data Set of 110 Hippocampi

The MIRIAD data set is used as the atlas data set. It is a database of volumetric MRI brain scans of patients suffering from Alzheimer’s disease and healthy elderly people. The data set is publicly available (www.ucl.ac.uk/drc/research/miriad) in anonymised form to aid researchers in developing new techniques for the analysis of serially acquired MRI. The atlas data set consists of 55 subjects who were recruited from the Cognitive Disorders Clinic at The National Hospital for Neurology and Neurosurgery, into a longitudinal neuroimaging study. All subjects underwent clinical assessment including the Mini-Mental State Examination (MMSE) [32]. All subjects gave written informed consent to take part in this study. Imaging data were used to create an average atlas using the groupwise registration algorithm described in 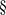 1.4 and in the parameter optimization process in 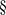 2.1. Subjects included 36 clinically diagnosed probable AD patients and 19 age-matched healthy controls. All patients fulfilled standard NINCDS/ADRDA criteria [33] for the diagnosis of probable AD. Subject demographics can be seen in Table 1. T1-weighted volumetric MR brain scans were performed on the same 1.5-T Signa unit (General Electric, Milwaukee), using an inversion recovery prepared fast SPGR sequence and a 256×256 image matrix with the field of view being 18 cm (acquisition parameters: repetition time = 15 ms; echo time = 5.4 ms; flip angle = 15°; inversion time = 650 ms). The volumetric scans were reconstructed as 124 contiguous 1.5-mm coronal images. T1-weighted volumetric scans were evaluated by one rater. All scans were N3 corrected [34] and bias correction was performed.

**Table 1:** Subject demographics in control and probable AD subjects used for parameter optimization. Mean (SD) unless specified otherwise.

	Control (n = 19)	AD (n = 36)
Age, years	68.7 (7.0)	69.6 (7.3)
Gender male (%)	9 (47%)	14(39%)
MMSE at baseline, /30	29.5 (0.7)	19.4 (4.1)

The left and right hippocampal regions were manually segmented by an expert segmentor S. The segmentation protocol is presented in the Appendix S1. The intra-rater variability measured by an ICC is 0.98. The left hippocampal segmentations from all 55 subjects are flipped along the mid-sagittal plane. This flipping effectively doubles the size of the data set by allowing, for example, the left hippocampus of a target image to be matched to the right hippocampus in the atlas data set. Therefore, the final atlas data set consists of 110 hippocampal images.

### 1.8 ADNI Data Set of 30 Subjects

Data used in the preparation of this article were obtained from the Alzheimer’s Disease Neuroimaging Initiative (ADNI) database (www.adni.loni.ucla.edu). ADNI was launched in 2003 by the National Institute on Aging (NIA), the National Institute of Biomedical Imaging and Bioengineering (NIBIB), the Food and Drug Administration (FDA), private pharmaceutical companies and non-profit organizations, as a 5-year public-private partnership. The aims of ADNI included assessing the ability of imaging and other biomarkers to measure the progression of mild cognitive impairment (MCI) and early Alzheimer’s disease (AD).

The Principal Investigator of this initiative is Michael W. Weiner, MD, VA Medical Center and University of California - San Francisco. ADNI is the result of efforts of many co-investigators from a broad range of academic institutions and private corporations, and subjects have been recruited from over 50 sites across the U.S. and Canada. The initial goal of ADNI was to recruit 800 adults, ages 55 to 90, to participate in the research, approximately 200 cognitively normal older individuals, 400 people with MCI and 200 people with early AD. For up-to-date information, see www.adni-info.org.

The 30 ADNI subjects (10 AD, 10 MCI and 10 controls) used for method validation consist of preprocessed baseline volumetric T1-weighted MR images acquired using 1.5T scanners (GE Healthcare, Philips Medical Systems or Siemens Medical Solutions) at multiple sites from the ADNI website. Representative imaging parameters were TR = 2400 ms, TI = 1000 ms, TE = 3.5 ms, flip angle = 8°, field of view = 240×240 mm and 160 sagittal 1.2 mm-thick slices and a 192×192 matrix yielding a voxel resolution of 1.25×1.25×1.2 mm^3^, or 180 sagittal 1.2 mm-thick slices with a 256×256 matrix yielding a voxel resolution of 0.94×0.94×1.2 mm^3^. The details of the ADNI MR imaging protocol are described in [35], and listed on the ADNI website (www.loni.ucla.edu/ADNI/Research/Cores/). Each scan underwent a quality control evaluation at the Mayo Clinic (Rochester, MN, USA). Quality control included inspection of each incoming image file for protocol compliance, clinically significant medical abnormalities, and image quality. The T1-weighted volumetric scans that passed the quality control were processed using the standard ADNI image processing pipeline, which included post-acquisition correction of gradient warping [36], B1 non-uniformity correction [37] depending on the scanner and coil type, intensity non-uniformity correction [34] and phantom based scaling correction [38] with the geometric phantom scan having been acquired with each patient scan.

Table 2 shows the clinical and demographic data of the 30 ADNI subjects. The same expert segmentor S as previously mentioned manually delineated the left hippocampus of those subjects. A segmentor S2 also manually delineated the left hippocampus on the same baseline images. The segmentation protocol is presented in the Appendix S1. The inter- and intra-rater reliability correspond to a Dice’s similarity index of 0.93 and 0.96 respectively.

**Table 2:** Subject demographics in set of 30 labelled randomly selected subjects used for method validation.

	Control (n = 10)	MCI (n = 10)	AD (n = 10)
Age, years	78.6 (5.4)	75.3 (8.8)	77.2 (6.8)
Gender male (%)	6 (60%)	7 (70%)	7 (70%)
MMSE, /30	29.5 (0.7)	27.4 (1.8)	27.0 (2.7)

Mean (SD) unless specified otherwise.

## Experiments

### 2.1 Optimizing Manifold Learning Parameters Using a Manually Segmented Data Set of 110 Atlases

A leave-one-out approach that excludes both the left and right hippocampi of the target image from the library of 110 atlases is used to optimize the parameters for each manifold learning technique. The following 4-step procedure is repeated for each atlas 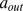 in the library. (i) After excluding 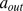 and its flipped image from the library, an average atlas 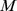 is built from the remaining 108 images in the data set. Distances between remaining atlases are computed based on the non-rigid transformations that align them to 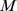 as described in 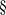 1.4. (ii) A manifold is computed from the remaining 108 atlases. (iii) The embedding is extended with 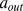. Distances between 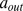 and the remaining atlases are derived by registering it to 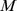 and performing subtraction of displacement fields. (iv) Its 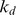-nearest neighbours are identified on the manifold using the L2 norm and combined in STAPLE to yield an estimated segmentation of 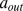.

Dice’s similarity index [39] is used for evaluation and is computed by measuring the overlap between the estimated segmentation and the manual segmentation. Dice’s similarity index is defined as 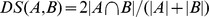, where A is the set of voxels in the automated region and B is the set of voxels in the manual region. A Dice’s similarity index is calculated for each 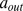 and a mean Dice’s similarity index 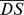 is calculated by averaging all 110 scores.

There is no defined procedure to establish the number of dimensions 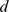 of a learned manifold, and the number of neighbours 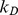 to build the connected graph in the high-dimensional space is often determined empirically. Results are evaluated for 3 different techniques: Isomap, LLE and LEM with dimension 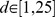 and a neighbourhood number of 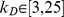 for each manifold technique. Using STAPLE with a MRF strength of 0.2, segmentations are generated by combining the closest 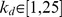 neighbours to 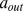 in the lower dimensional space. For LEM, 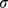 is set to 1. A 4D matrix of mean Dice’s similarity indexes is then computed with the following axes: manifold type 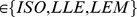, 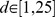, 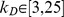, and 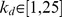. The coordinates in this matrix that give the highest 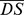 indicate the best manifold learning technique with optimized parameters for this data set.

In order to compare atlas selection *with* manifold learning to atlas selection *without* manifold learning, we also compute the results given by a plain 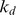-nearest neighbour selection in the high-dimensional space 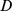. For each 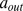, its 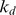-nearest neighbours in the high-dimensional space 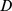 are computed using the metric defined in 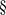 1.4 and combined in STAPLE to yield an estimated segmentation. As before, a Dice’s similarity index is calculated for each 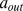 and a mean Dice’s similarity index 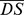 is calculated by averaging all 110 scores. We refer to this selection method as BASE and results are computed for 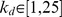.

### 2.2 Method Validation Using a Manually Segmented Data Set of 30 ADNI Subjects

For method validation, the left hippocampus in the baseline images of 30 randomly selected subjects in the ADNI database (10 AD, 10 MCI and 10 controls) were segmented. Those images differ from the MIRIAD data set of atlases used for parameter optimization. The atlas data set of 110 images is used to segment each of the ADNI target images. The optimal parameters determined in 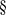 2.1 are used to generate left hippocampal regions. Since the right hippocampus segmentations for this set of 30 subjects were not available, we only evaluate the accuracy of our method on the left hippocampus.

## Results

### 3.1 Results from Method Optimization Using a Manually Segmented Data Set of 110 Atlases

**Figure 1:**
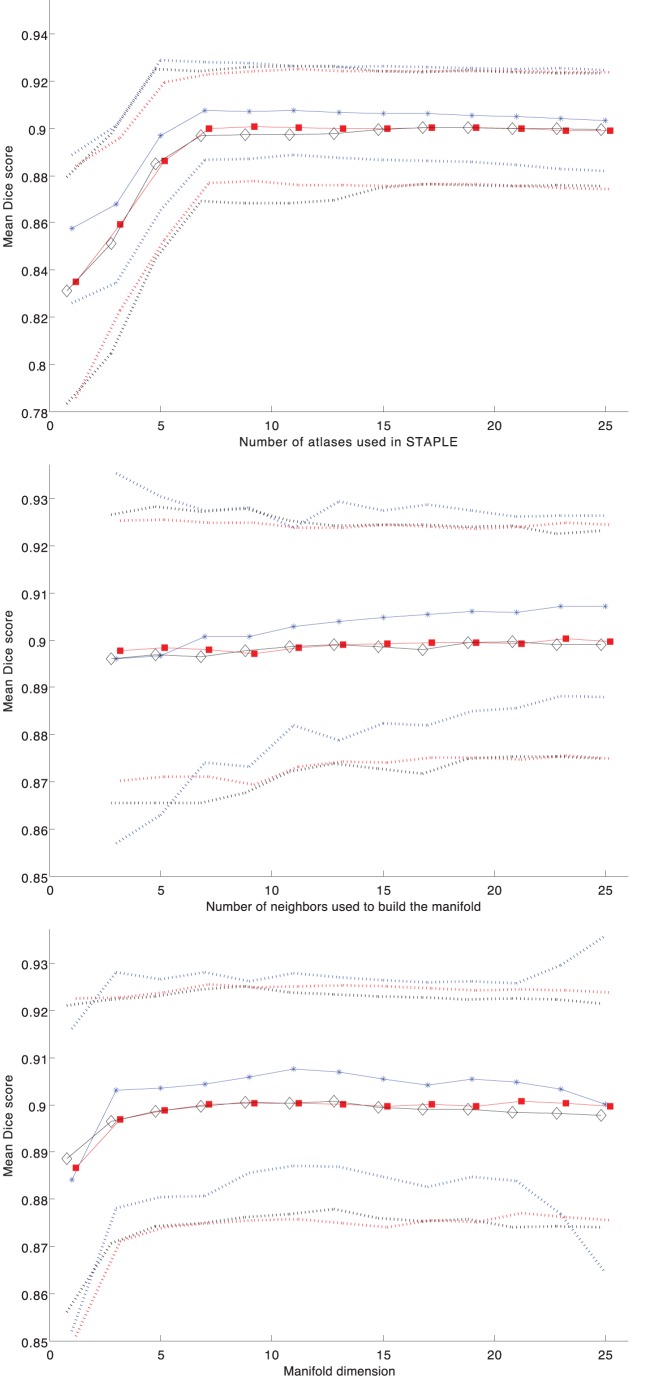
Mean Dice’s similarity index computed for 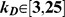, 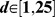, 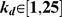. Locally Linear Embedding is in blue, Isomap is in red and Laplacian Eigenmaps is in black. Solid lines represent the mean Dice’s similarity index, doted lines represents the standard deviation. Mean Dice’s similarity index against: (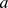) the number of atlases fused in STAPLE (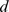 and 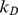 fixed to best parameters), (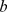) the neighbourhood size 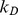 in computing the manifold (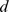 and 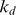 fixed to best parameters), and (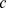) the manifold dimension 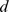 (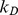 and 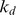 fixed to best parameters).

The best combination of manifold learning technique and parameters is Locally Linear Embedding with a manifold dimension of 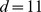, a neighbourhood size 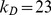 and combining the top 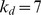 matches in STAPLE, giving a mean (SD) Dice’s similarity index 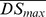 of 0.9077 (0.0211). In contrast, Isomap and Laplacian Eigenmaps resulted in Dice’s similarity indexes of 0.8995 (0.0228) and 0.8971 (0.0245) with 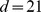, 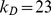 and 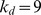 and 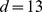, 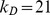 and 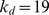 respectively. Each graph in Figure 1 shows the mean Dice’s similarity index for each manifold learning technique when 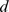, 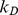 and 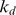 are fixed to their respective optimal parameters. It is interesting to note that all 3 manifold learning techniques result in a very high mean Dice’s similarity index (>0.89). Using a 2-tailed paired 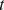-test, Locally Linear Embedding gives a significantly (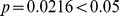 and 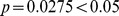) higher average Dice’s similarity index compared to Isomap and Laplacian Eigenmaps, whereas the difference between Isomap and Laplacian Eigenmaps is not statistically significant (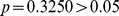). The accuracy achieved by fusing multiple segmentations quickly rises to a maximum and then gradually declines as the number of segmentations increases. This is in line with results published in [5] and [7] : the gradual decline corresponds to adding dissimilar images into the combination process, resulting in segmentation errors. The accuracy also flattens out for manifolds of 3 or more dimensions. This suggests that our data set of hippocampi can be described mostly by 3 main modes of variation, and this is consistent across all manifold learning techniques presented. The number of neighbours 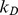 used to build the connected graph has little effect on the accuracy when using Isomap and Laplacian Eigenmaps. In contrast, increasing 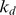 increases the accuracy achieved with Locally Linear Embedding.

Table 3 compares the mean Dice’s similarity index (SD) obtained by selecting atlases with manifold learning and using the BASE method. The results show that all 3 manifold learning selection methods significantly outperform 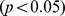 the plain selection method.

**Table 3:** Mean Dice’s similarity indexes 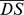 (SD) obtained with manifold learning selection (LLE, ISO, LEM) and plain selection (BASE).

	LLE	ISO	LEM	BASE
	*d* = 11, *k*_D_ = 23, *k_d = _*7	*d* = 21, *k*_D_ = 23, *k_d_* = 9	*d* = 13, *k*_D_ = 21, *k*_d_ = 19	*k*_d_ = 9
Mean DS	0.9077	0.8995	0.8971	0.8756
(SD)	(0.0211)	(0.0228)	(0.0245)	(0.0219)
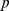 -value	LLE vs.	ISO vs.	LEM vs.	BASE vs.
	ISO, *p* = 0.0216	LLE, *p* = 0.0216	LLE, *p* = 0.0275	LLE, *p* = 0.0056
	LEM, *p* = 0.0275	LEM, *p* = 0.3250	ISO, *p* = 0.3250	ISO, *p* = 0.0137
	BASE, *p* = 0.0056	BASE, *p* = 0.0137	BASE, *p* = 0.0204	LEM, *p* = 0.0204

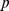
-values comparing each approach with each other are reported.

**Figure 2:**
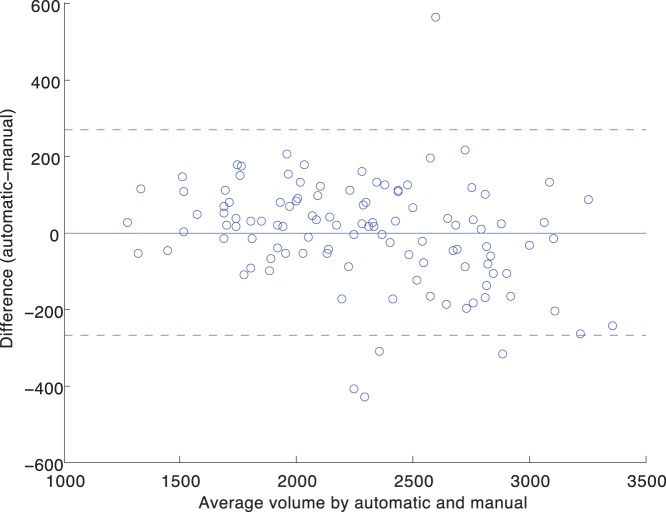
Bland-Altman plot. Each point corresponds to an hippocampal segmentation. The difference between automatic and manual estimates is plotted against their average. The solid horizontal line corresponds to the average difference, and the dashed lines are plotted at average +/−1.96 standard deviations of the difference.

**Figure 3:**
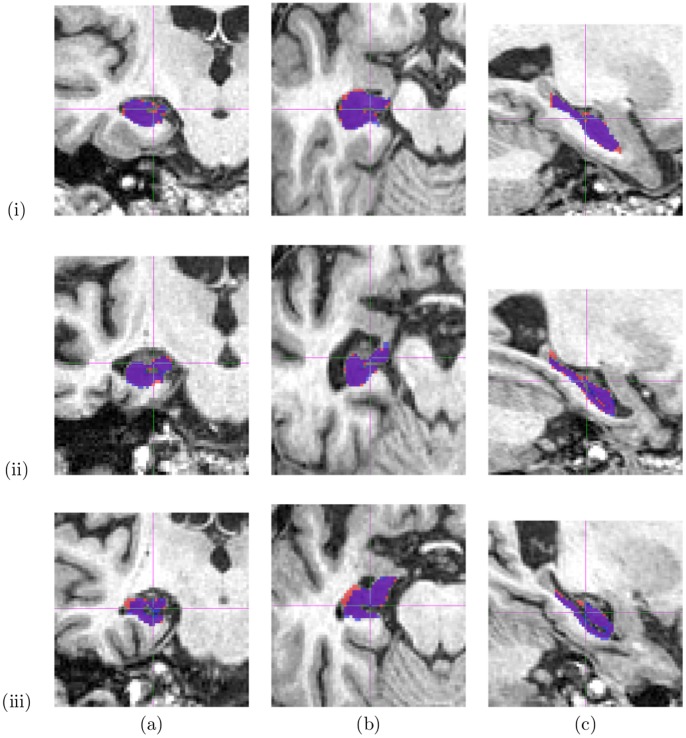
Hippocampal segmentation: automated (blue) vs manual (red). Overlapping area in purple. Row: (i) High case (Dice = 0.9398), (ii) Typical case (Dice = 0.9073), (iii) Low case (Dice = 0.8614). Column: (a) Coronal view, (b) Sagittal view, (c) Axial view.

Table 4 shows the mean (SD) of the manual and automated hippocampal volumes. The automated volumes were computed using Locally Linear Embedding with the optimized parameters. The mean (SD) of differences between the manual and automated hippocampal volumes by baseline diagnostic group was 27 (129) mm^3^ (automated<manual) for controls and −12 (150) mm^3^ (automated>manual) for AD subjects. In order to test the validity of our method, we compare the proposed method to a state-of-the-art method for hippocampus segmentation based on a similar atlas library approach [7]. Using the same library of 110 hippocampus images and optimal parameters defined in [7], a similar leave-one-out method is performed. The mean Dice’s similarity index was 0.8955 (0.0172) compared to 0.9077 (0.0211) in our method. Even though these values differ by 0.01 point only, the difference is statistically significant (p<0.001). Figure 2 plots the volume correlation between the manual segmentation and our automatic segmentation method. The volume differences between manual segmentation and automatic segmentation are similar to zero-mean random noise. Figure 3 shows an example of segmentation obtained with our method.

**Table 4:** Mean (SD) of the volumes (in mm^3^) in the left hippocampus in the baseline images of the atlas library of 110 images used to assess optimal methods and parameters.

	Control (n = 19)	AD (n = 36)
Manual (SD)	2749 (273)	2054 (424)
Automated (SD)	2722 (249)	2066 (387)
Man vs Auto mean of difference ( 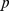 -value)	27 (p = 0.19)	−12 (p = 0.14)
SD of differences	129	150

Overall, these results show that registering atlases that have been selected by manifold learning (i.e. selection in the lower-dimensional space) produces accurate and robust segmentation in the framework of multi-atlas based segmentation and gives better results compared to atlas selection without manifold learning (i.e. selection in the high-dimensional space). Also, given our data set of atlases, Locally Linear Embedding gives significantly better results than Isomap and Laplacian Eigenmaps.

### 3.2 Results from Method Validation Using a Manually Segmented ADNI Data Set of 30 Subjects

**Figure 4:**
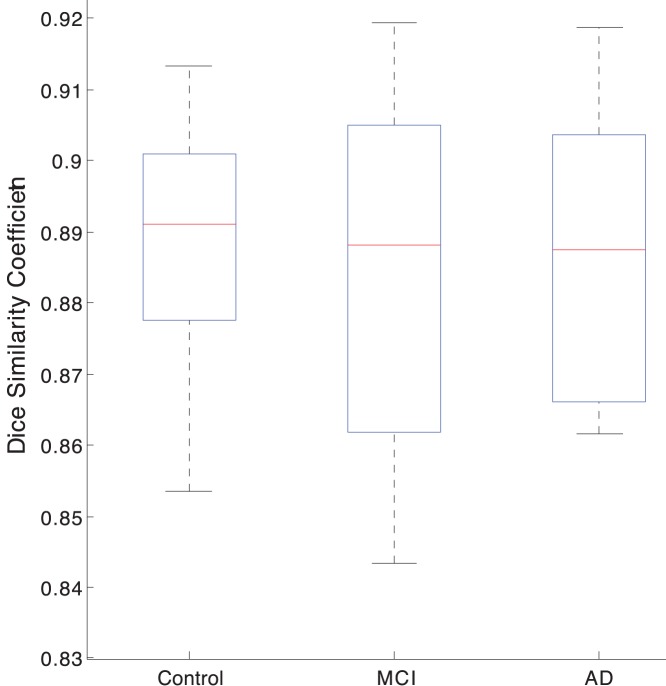
Average Dice’s similarity index for NC, MCI and AD group obtained by fusing top 7 atlases with STAPLE. Atlases were selected with manifold learning.

We use Locally Linear Embedding with the optimal parameters found in 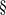 3.1 to generate automatic segmentation of the 30 ADNI subjects. The mean (SD) Dice’s similarity indexes of the left hippocampus segmentations of the baseline ADNI images are 0.887 (0.020) for controls, 0.886 (0.025) for MCI, 0.878 (0.038) for AD and 0.883 (0.028) across the three groups. These are summarized in Figure 4. The difference in accuracy compared to the previous experiment can be explained by the fact that the atlases and the 30 ADNI subjects belong to different data sets. Also the high shape variability and the possible presence of cysts in the hippocampus can explain lower scores in AD subjects. Table 5 shows the means (SD) of the manual and automated hippocampal volumes. The mean (SD) of differences in the manual and automated hippocampal volumes by baseline diagnostic group are −111 (168) mm^3^ for controls, −3 (155) mm^3^ for MCI, and −24 (130) mm^3^ for AD subjects with automated volumes higher than manual volumes in all the three groups. Overall, the mean (SD) of differences in the manual and automated hippocampal volumes is −45 (154) mm^3^. We also calculate the effect size 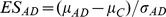 and 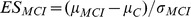 in Table 6, where 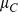, 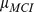, 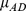 are the average volumes in the control, MCI and AD groups respectively, and 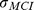, 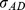 are the standard deviations in the MCI and AD groups respectively.

**Table 5:** Mean (SD) of the volumes (in mm^3^) in the left hippocampus in the baseline images of the labelled ADNI data set of 30 images for method validation.

	Control (n = 10)	MCI (n = 10)	AD (n = 10)
Manual (SD)	2531 (336)	2331 (410)	1994 (478)
Automated (SD)	2642 (360)	2334 (431)	2018 (387)
Man. vs Auto. mean of diff. ( 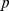 -value, paired t-test)	−111 (p = 0.33)	−3 (p = 0.47)	−24 (p = 0.29)
SD of differences	168	155	130

**Table 6:** Effect size.

	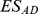	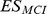
Manual (SD)	−1.124	−0.490
Automated (SD)	−1.614	−0.720

### Conclusions

We compared Isomap, Locally Linear Embedding and Laplacian Eigenmaps for the selection of atlases to use in multi-atlas segmentation of the hippocampus of normal controls and patients with Alzheimer’s disease in MR images.

We found that Locally Linear Embedding generated the best hippocampal segmentation (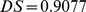) on a leave-one-out experiment using our data set of 110 atlases. The mean volumes and SDs of the generated segmentations were similar to those produced using manual segmentation. Overall, the mean difference between our automated volumes and the manual measurements was 7.5 mm^3^ or around 0.01% of the mean of all volumes. We found good accuracy of our method on unseen data, achieving a mean Dice’s similarity index of 0.883 (0.028) when comparing the automated and manual segmentations of a set of 30 subjects (10 AD, 10 MCI and 10 controls). Overall, the mean (SD) of differences in the manual and automated hippocampal volumes was 45 (154) mm^3^ with manual<automated.

Our results are consistent with those in [40]. They found that large number of 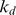-nearest neighbours leads to higher Dice’s similarity index for large database size M and that Dice’s similarity index decreases as 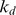 approaches the value of M. In our study, the Dice’s similarity index quickly rises to a maximum when the number of 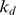-nearest neighbours increases for all the manifold learning techniques. The Dice’s similarity index then gradually declines as the number of 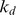-nearest neighbours increases.

Not only is the choice of manifold learning important but also the parameters used to compute the embedding. For instance, most studies have represented the embedding with 2 or 3 dimensions as it enables spacial visualization of the embedding. However the optimal embedding could have been of higher dimensions. Indeed, in our study, we found that the best results arose when using 11 dimensions. Also all manifold learning techniques presented in this paper require the choice of a neighbourhood size either for the calculation of the geodesic distance in Isomap, or reconstructing a data point with its closest points in Locally Linear Embedding or Laplacian Eigenmaps. The choice of the optimal dimension and best parameters is often made empirically.

The results showed that selection of atlases with manifold learning is beneficial in the framework of multi-atlas based segmentation. The optimal accuracy can be found by fine tuning the manifold learning process. It also turned out that our atlas data set of hippocampi can be described by 3 main modes of variation regardless of the manifold learning technique used.

We found that Locally Linear Embedding gave best results for our data set of the hippocampus but it might not yield optimum results for a different anatomical structure. There is no consensus on which manifold learning technique to use for a given data set. A legitimate question that arises is which manifold learning algorithm is best suited for which data set. As demonstrated in this study, different manifold learning techniques produce different low-dimensional embeddings even for the same data set. This can be explained by the fact that the cost function to optimize associated with a manifold learning technique differs from one method to another.

The lower Dice’s similarity index obtained when segmenting the 10 AD subjects from the ADNI data may also illustrate the issue of manifold sampling. Since the manifold is directly learned from points (i.e. images) in the data set, the sampling of the manifold is highly correlated with the density of points in the high-dimensional space. For example, if certain areas in the high-dimensional space are too sparse, the resulting manifold is likely to be a poor approximation of the true manifold structure. Since the atlas data set did not contain any MCI subjects, the manifold derived from this atlas data set is not representative of a population containing NC, MCI and AD subjects. It would have been preferable to derive a manifold from NC only subjects in the atlas data set to segment the 10 NC from the ADNI data set, and similarly for the 10 AD in the ADNI data set.

An important aspect in manifold learning is the metric used to relate pairs of images in the high-dimensional space. The most commonly used metrics are based on voxel intensity such as the Euclidean distance, cross correlation or mutual information. Similarly to [17] and [14], we used a metric derived from non-rigid transformation. In theory, the metric used should reflect the information relating pairs of images [24], [41]. However, there is currently no research investigating the influence of the metric on the resulting embedding. In the future, we are planning to compare the effects of several metrics such as the geometric median and the geodesic estimation proposed by [42] and [43] respectively on low-dimensional embeddings.

We have obtained one of the best accuracies reported to date for automated hippocampal segmentation when compared with gold standard manual segmentations from a set of 30 randomly chosen subjects (10 AD, 10 MCI and 10 controls) from ADNI. Our Dice’s similarity index is equal to 0.88 with the previous highest Dice’s similarity indexes (N = number of hippocampi in the study) being 0.86 (N = 14) [44], 0.83 (N = 60) [45], 0.81 (N = 100) [46], 0.86 (N = 54) [47], 0.87 (N = 30) [48], 0.88 (N = 5) [49] (from a cohort of 2 year old children), 0.86 (N = 40) [50], 0.85 (N = 30) [51], 0.86 (N = 40) [52], 0.83 (N = 550) [5], 0.89 (N = 160) [53], 0.89 (N = 30) [7], 0.89 (N = 120) [8] and 0.85 (N = 364) [12]. Our intra-rater variability corresponds to a Dice’s similarity index of 0.96. Comparing this to the results from using our automatic method with different training and test data (0.88) suggests that the method has not been over-trained, and that there is potential to improve it further.

Overall, our technique is most similar to that reported by [12]. However it fundamentally differs in the following ways: (i) [12] used a similarity measure derived from voxel intensities, whereas we used a metric derived from registration. (ii) We embedded target images using the out-of sample extension instead of embedding all images in a single manifold. This method effectively scales with the number of atlases and not the number of images to segment. (iii) We used STAPLE as a fusion method, whereas statistical voxel classification and graph cuts was used in [12].

We developed a suitable method for segmenting large data sets by extending the manifold with an out-of-sample image. Indeed, in our method: (i) the low-dimensional manifold learned from the space spanned by the set of atlases, (ii) the average atlas M and (iii) the registrations between the atlases and M are precomputed and stored, thus making our method very computationally efficient. We only need to perform one non-rigid registration between M and a new unseen target image 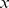 to select its most similar images from the atlases. This method is therefore scalable and extremely computationally efficient, making it suitable for segmenting large data sets and for clinical use. For instance, in the context of radiotherapy treatment, we are planning to apply our method to CT images of head and neck, where segmentations of tumor regions and organs at risk (such as the parotid glands and lymph nodes) show low agreement within and between raters due to poor boundary definition on CT images.

To conclude, manifold learning produces accurate segmentation in the framework of multi-atlas segmentation by improving atlas selection. Our method shows that Locally Linear Embedding gave better results in our experiments, however using a different atlas data set with a different density distribution will probably require the re-computation of the optimized parameters and manifold for segmentation.

## Supporting Information

Appendix S1 Hippocampus segmentation protocol. (PDF)
