# Supplementary material for: Using Manifold Learning for Atlas Selection in Multi-Atlas Segmentation
Source: PLoS One. 2013 Aug 2;8(8):e70059. doi: 10.1371/journal.pone.0070059 (PMC3732273; doi:10.1371/journal.pone.0070059)
Supplement: Appendix S1 — (PDF) [file pone.0070059.s001.pdf]

Appendix S1. Hippocampus segmentation protocol.

All segmentations were performed using MIDAS software which allows images to be reconstructed in 3D allowing segmentation and viewing in coronal, axial and sagittal planes [54]. Brain regions were segmented using a previously described method [54], and all images were registered into MNI 305 atlas space [55] using nine degrees of freedom and then applying only six of these (translations and rotations of the scans but no scalings) to ensure all images were in a similar orientation. Each image was therefore resampled to produce isotropic voxels. Hippocampi were delineated for each subject by an experienced rater (J.B.). Rater J.B. has over 5 year experience of hippocampal segmentation having segmented approximately one thousand hippocampi. Intra-subject reliability measured by an ICC was 0.98, based on same-scan analysis of 20 subjects segmented twice.

The hippocampus was always measured on the right-hand side of the presented image with the investigator blinded to the subject's name, diagnosis, and left-right orientation of the scans. Each hippocampus took approximately 45 min to delineate (1.5 h per scan). The hippocampus was defined as including the dentate gyrus, the hippocampus proper, the subiculum and the alveus. Measurements were taken from every coronal slice from the posterior to anterior boundaries using a standard neuroanatomical atlas [56]. The posterior limit of the hippocampus was defined as the coronal slice where the longest length of the crus of the fornix was seen [57]. The hippocampus was bounded superiorly, medially and laterally by CSF and inferiorly by the white matter of the subiculum. The head of the hippocampus was delineated from the amygdala by inclusion of the alveus, which is best seen as a band of high signal intensity on the sagittal sections. A minimum threshold of 70% of mean brain intensity was applied to improve consistency in the exclusion of voxels which were predominantly CSF.
